# Monitoring the Changes of Material Properties at Bone-Implant Interface during the Healing Process In Vivo: A Viscoelastic Investigation

**DOI:** 10.1155/2017/1945607

**Published:** 2017-03-08

**Authors:** Hsiang-Ho Chen, Wei-Yi Lai, Tze-Jian Chee, Ya-Hui Chan, Sheng-Wei Feng

**Affiliations:** ^1^School of Biomedical Engineering, College of Biomedical Engineering, Taipei Medical University, 250 Wu-Hsing Street, Taipei, Taiwan; ^2^Department of Molecular Science and Engineering, National Taipei University of Technology, No. 1, Sec. 3, Zhongxiao E. Rd., Taipei, Taiwan; ^3^School of Dentistry, College of Oral Medicine, Taipei Medical University, 250 Wu-Hsing Street, Taipei, Taiwan; ^4^School of Oral Hygiene, College of Oral Medicine, Taipei Medical University, 250 Wu-Hsing Street, Taipei, Taiwan

## Abstract

The aim of this study was to monitor the changes of viscoelastic properties at bone-implant interface via resonance frequency analysis (RFA) and the Periotest device during the healing process in an experimental rabbit model. Twenty-four dental implants were inserted into the femoral condyles of rabbits. The animals were sacrificed immediately after implant installation or on day 14, 28, or 56 after surgery. Viscoelastic properties at bone-implant interface were evaluated by measuring the implant stability quotient (ISQ) using RFA and by measuring the Periotest values (PTVs) using the Periotest device. The bone/implant specimens were evaluated histopathologically and histomorphometrically to determine the degree of osseointegration (BIC%). The BIC% values at different time points were then compared with the corresponding ISQ values and PTVs. The mean ISQ value increased gradually and reached 81 ± 1.7 on day 56, whereas the mean PTV decreased over time, finally reaching −0.7 ± 0.5 on day 56. Significant correlations were found between ISQ and BIC% (*r* = 0.701, *p* < 0.001), PTV and BIC% (*r* = −0.637, *p* < 0.05), and ISQ and PTV (*r* = −0.68, *p* < 0.05). These results show that there is a positive correlation between implant stability parameters and peri-implant-bone healing, indicating that the RFA and Periotest are useful for measuring changes of viscoelastic properties at bone-implant interface and are reliable for indirectly predicting the degree of osseointegration.

## 1. Introduction

The success of dental implants depends on the stability of the implant, the quality of local bone, surgical skills, and patient factors [1, 2]. Implant stability plays an important role in successful osseointegration [3], which is defined as the direct structural and functional connection between ordered living bone and the surface of a load-carrying implant [4]. Recently, implant stability has been shown to be a useful predictor and measurement parameter of osseointegration in both clinical and experimental studies [5–9].

Implant stability occurs at two different stages [10]. Primary implant stability is achieved when the implant interlocks mechanically with the alveolar bone. Approximately 2–4 weeks after implant placement, primary implant stability is gradually replaced by secondary implant stability, which is obtained and maintained by the continuous regeneration of new bone and bone apposition and remodeling around the implant [5, 9–12]. Several methods and techniques have been developed in recent years to measure and monitor the changes in dental implant stability [13, 14].

Resonance frequency analysis (RFA) and the Periotest device (Siemens AG, Bensheim, Germany) are two widely used methods for noninvasively measuring dental implant stability at different surgical stages and during follow-up observations [14–18]. RFA measures resonance frequency, defined as the peak of the frequency-amplitude plot, through a piezoceramic transducer attached to the implant fixture. These vibrational signals are then converted into a value representing implant stability or stiffness at the bone-implant interface [19]. Osstell™ (Integration Diagnostics AB, Göteborg, Sweden), a commercially available RF device, converts the resonance frequency signals measured in kHz (range, 5 to 15 kHz) into implant stability quotient (ISQ) values ranging from 1 to 100 [11]. Higher ISQ values are indicative of greater implant stability. Clinical and experimental studies have demonstrated that RFA is a reliable technique for assessing osseointegration and evaluating prognosis [14, 16].

The Periotest device is designed to evaluate tooth mobility and implant stability based on damping capacity assessment [20]. This device electronically drives a metallic rod to strike the tooth or implant and calculates the contact time between the tapping rod and the tested subject. The detected contact time is converted into a unique value called the Periotest value (PTV), which ranges from −8 to 50. Lower values are indicative of greater rigidity of objects, which can be used to estimate bone healing status at the implant-bone interface.

Although the Osstell and Periotest devices are widely used in daily dental practice, the reliability and validity of these two methods are still questioned [8, 13]. In addition, studies have also suggested that the individual measurement of implant stability using RFA or Periotest should be performed with caution and used in combination with other objective methods or clinical parameters [8, 13, 21]. This is because there are controversies regarding the correlation between implant stability parameters (ISQ and PTV values) and histomorphometric data [8, 21–23].

Some animal studies have demonstrated poor correlation between ISQ values and histomorphometric data [23–26], whereas other animal and clinical studies have shown a positive correlation between ISQ values and histomorphometric data [22, 27–29]. In addition, Jun et al. demonstrated no significant correlation between PTV values and BIC% values in a human fresh cadaver study [21]. In contrast, Oh et al. reported that the values obtained from the Periotest device strongly correlate with the degree of osseointegration in dogs [28].

Although RFA and the Periotest devices are used to detect implant stability and determine the healing status at the implant/bone interface, the correlations between implant stability parameters and histomorphometric data during the healing process are still controversial and have not been definitively established. Therefore, the purpose of this study was to evaluate the relationship between ISQ, PTV, and BIC values in a rabbit model.

## 2. Materials and Methods

### 2.1. Animals and Ethics

The study protocol was approved by the institutional Animal Care and Use Committee of the Taipei Medical University, Taipei, Taiwan (approval number LAC-2016-0174). All animal experimental procedures were carried out according to the ethical regulations of the international guiding principles for the care and use of laboratory animals.

### 2.2. Experimental Animals and Surgical Procedures

Animals in this study comprised 12 adult male New Zealand White rabbits aged 10 months and weighing 3.0–3.5 kg. All rabbits were housed in individual cages and provided ad libitum access to water and food under standard laboratory conditions at the Laboratory Animal Center of the Taipei Medical University. The flat medial femoral condyles of both legs were selected as the surgical sites. A total of 24 Brånemark® dental implants (Nobel Biocare AB, Göteborg, Sweden) measuring 3.75 mm in diameter and 10 mm in length were inserted by the same experienced surgeon.

**Figure 1:**
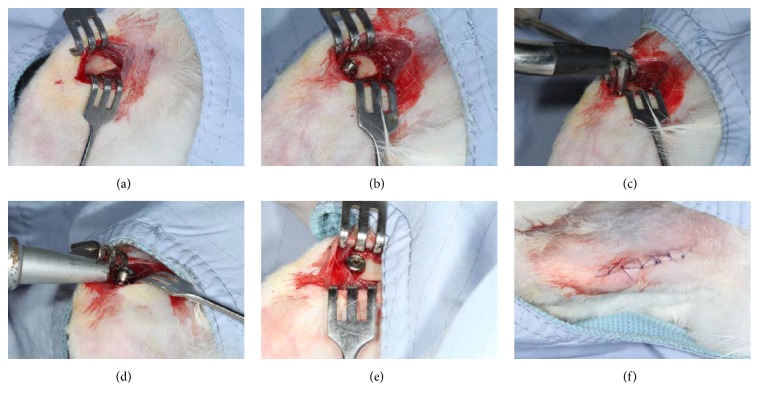
Clinical images of the surgical and experimental procedures. (a) Surgical exposure of the medial surface of the proximal condyles. (b) Implant installation. (c) ISQ values were measured using the Osstell device. (d) PTV values were measured using the Periotest device. (e) A cover screw was installed into the implant fixture. (f) Complete wound closure.

The rabbits were anesthetized by intramuscular injection of tiletamine-zolazepam (Zoletil 50, Virbac, Carros Cedex, France) at a dose of 15 mg/kg. The surgical sites were shaved, washed, and disinfected with iodine antiseptic solution and isolated with surgical towels. Local anesthesia (1.8 mL of 2% with 1 : 100,000 epinephrine) was administered intramuscularly at the surgical site of each leg. After making an incision of approximately 3 cm in length, the muscle was bluntly dissected and the periosteum was reflected using a periosteal elevator to expose the flat bone surfaces at the medial aspect of the femoral condyle (Figure 1(a)). The implant recipient site was sequentially prepared according to the protocol recommended by the manufacturer. At first, a round bur was used to mark the implant site and penetrate the cortical layer. Subsequently, twist drills with diameters of 2.0, 2.4/2.8, 3.0, 3.2, and 3.4 mm were used to drill holes to a depth of 10 mm under generous saline cooling. Twenty-four cylindrical screw-type titanium dental implants (Brånemark, Nobel Biocare AB, Göteborg, Sweden) measuring 3.75 mm in diameter and 10 mm in length were inserted into the femoral condyles of both legs. All of the implants were inserted unicortically and threaded to the bone level (Figure 1(b)). Immediately after implant insertion, the implant stability of each tested implant was measured by the Periotest and the Osstell RFA devices (Figures 1(c) and 1(d)). After measuring the stability parameters, the cover screws were secured (Figure 1(e)) and the surgical sites were closed layer-by-layer with absorbable sutures (Vicryl® 4.0, Ethicon, Somerville, NJ, USA) (Figure 1(f)). All rabbits were then moved back to the recovery room and observed for any signs of wound dehiscence. Postoperative antibiotics (Baytril®, Bayer, Leverkusen, Germany) (5.0 mg/kg) and analgesics (Rimadyl®, Pfizer, New York, USA) (4.0 mg/kg) were injected intramuscularly for 3 days to prevent infection and control pain. The 12 animals were divided into four groups of three animals each. Animals in group A were sacrificed immediately after implant insertion (day 0) and animals in groups B-D were sacrificed at 14, 28, and 56 days after the operation, respectively.

### 2.3. Measurements of Implant Stability Parameters

A “SmartPeg” (Integration Diagnostics AB, Göteborg, Sweden), an aluminum metal rod with a magnet attached to its top, was screwed into each of the tested implants. The SmartPeg was manually tightened to approximately 5 Ncm according to the manufacturer's guidelines. The analyzer probe was then placed close to the SmartPeg in the same direction perpendicular to the long axis of the femur to standardize the experimental procedure. The SmartPeg is excited by a magnetic pulse generated by the measurement probe, which produces a vibrational signal that is detected by the handheld instrument. The resonance frequency measured by the Osstell system (Integration Diagnostics AB, Göteborg, Sweden) is expressed as an ISQ value ranging from 0 to 100. Three measurements were taken per implant and the mean value was recorded as the final ISQ value.

PTV values were measured using the Periotest device (Siemens AG, Bensheim, Germany). After the insertion of healing abutment (Brånemark RP Abutment, Nobel Biocare, Sweden) with 4 mm height into the implant, the PTV was measured 3 times along the long axis. According to the manufacturer's instructions, the metallic rod of the Periotest device was positioned perpendicular to the long axis of the tested healing implant, which was then tapped 20 times within a 5-second period. The measurements were made at the same time intervals as RFA measurements, namely, immediately after implant insertion (day 0) and then at 14, 28, and 56 days after the operation.

### 2.4. Preparation of Histological Specimens

Three rabbits were sacrificed at each time point by an overdose of pentobarbital. The femoral condyles containing the implants were harvested using a diamond circular saw and subsequently fixed in 10% formalin solution for 7 days. Whole implant-bone samples were then processed without decalcification for ground sectioning according to the previous studies [8, 23]. In brief, the specimens were dehydrated in a graded series of ethanol (70% to 100%) over a period of 24 hours at 5°C and defatted in xylene under vacuum. The specimens were then embedded in methacrylate-based resin (Technovit 9100; Heraeus Kulzer GmbH, Wehrheim, Germany) according to the manufacturer's instructions. Following polymerization, the embedded blocks were cut into slices parallel to the long axis of the implant using a rotary diamond-coated saw (AZ-CL40, Yeong Shin Hardware, Taipei, Taiwan) with coolant. The slices were glued to slides to prevent damage to the bone-implant interface. Subsequently, the ground sections were thinned to a final thickness of approximately 80 *μ*m using a series of abrasive papers (400, 600, 800, 1000, and 1200 *μ*m) in a grinding/polishing machine with running water.

### 2.5. Histomorphometrical Procedures

The sections were stained with 1% toluidine blue (Sigma-Aldrich Chemie GmbH, Buchs, Switzerland), which stains mineralized bone as violet blue and the osteoid as pale blue. The histological analysis of the implant/bone interface was performed under an optical microscope (Nikon, Alphaphot-2, YS-2, Tokyo, Japan) equipped with a Spot digital camera and software (Diagnostic Instruments, Inc., Sterling Heights, MI, USA) by an independent examiner. Histomorphometrical analysis was performed using the Image-Pro Plus 6.0 image analysis system (Media Cybernetics, Silver Spring, MD, USA). The percentages of bone-to-implant contact (BIC%) over the entire length of the implant were calculated by measuring the percentage of the distance of the mineralized bone in direct contact with the implant surface. All measurements were performed for both sides of the implant on three histological sections per implant. All calculations were performed using EXCEL software (Microsoft Corporation, Redmond, WA, USA).

### 2.6. Statistical Analysis

ISQ, PTV, and BIC data for each tested implant are expressed as mean values and standard deviations. One-way analysis of variance (ANOVA) with Tukey's honest significant difference test was performed to compare differences at each time point. The coefficient *r*^2^ was calculated to measure the correlation estimates and Pearson correlation coefficient was used to measure a significant association between ISQ, PTV, and BIC values. A *p* value < 0.05 was considered to indicate statistical significance. All statistical analyses were performed with the statistical package SPSS for Windows (Version 19, SPSS Inc., Chicago, IL, USA).

## 3. Results

### 3.1. Experimental Animal and Implant Outcome

None of the rabbits showed signs of inflammation or other adverse tissue reactions during the healing period. All surgical sites healed well and no swelling or redness was noted.

### 3.2. Implant Stability Parameters

**Figure 2:**
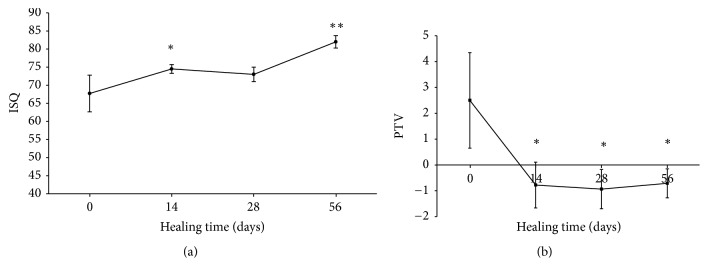
The healing curves plotted with (a) ISQ and (b) PTV values (^*∗*^*p* < 0.05; ^*∗∗*^*p* < 0.01).

The implant stability parameters determined by measuring ISQ and PTV are presented in Figures 2(a) and 2(b). As shown in Figure 2(a), the ISQ values continuously increased during the study period. The initial mean ISQ value was 67.7 ± 5. Then, the ISQ values increased to 74.5 ± 1.2 at day 14 (*p* < 0.05) and remained stable at day 28 (73 ± 2) (*p* < 0.05). No significant difference was observed between the groups at days 14 and 28. After 56 days of healing, the mean ISQ value (81.9 ± 1.7) was significantly higher than the initial mean ISQ value (67.7 ± 5) (*p* < 0.001). The mean ISQ value at final measurement was 20.9% higher than the initial ISQ value.

In contrast, the PTVs progressively decreased from day 0 to 56 days after implant installation (Figure 2(b)). The mean PTV was 2.5 ± 1.8 at day 0 and −0.7 ± 0.8 at day 14. After that, the PTVs remained stable on day 28 (−0.9 ± 0.7) and day 56 (−0.7 ± 0.5). There were statistically significant differences in PTVs at days 14, 28, and 56 (*p* < 0.05) compared with the initial PTV. However, there were no significant differences among PTVs at days 14, 28, and 56.

### 3.3. Histologic and Histomorphometrical Evaluations

**Figure 3:**
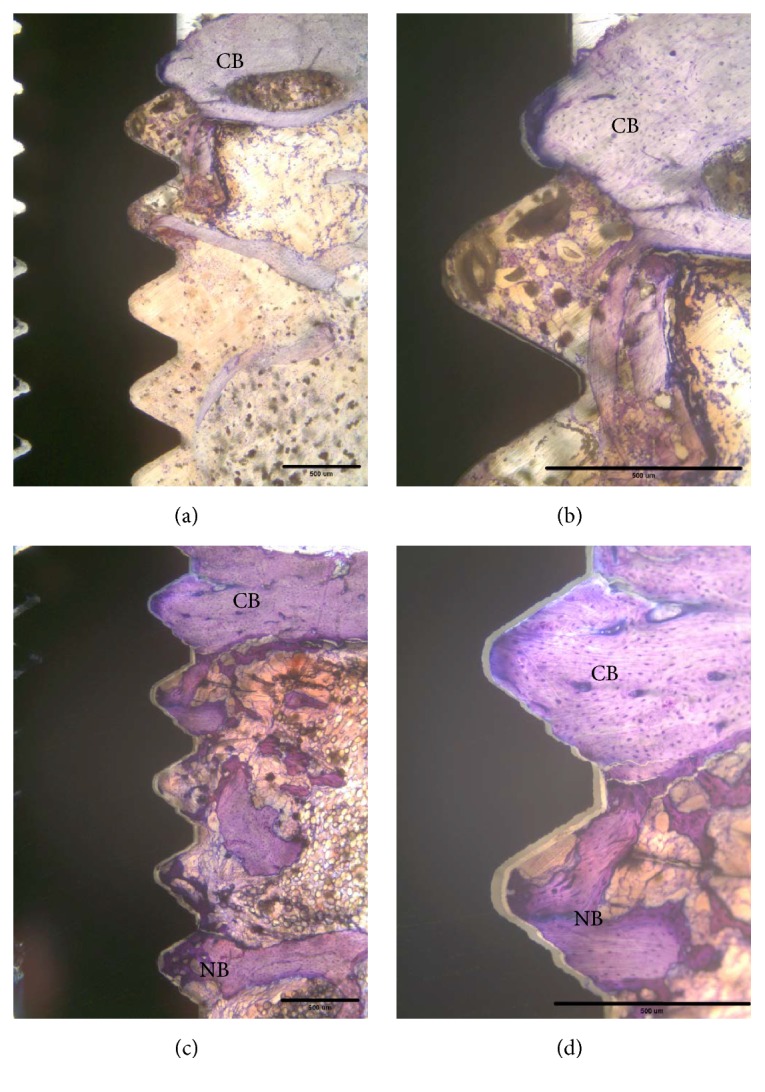
Histological findings of the tested implant after 0 days and 14 days of healing. (a) The implant is partly surrounded with original cortical bone (CB). No peri-implant new bone (NB) formation was observed in a bone marrow cavity. Original magnification ×2.5. (b) Original magnification ×10. (c) The implant is surrounded by cortical bone and newly formed bone in the bone marrow cavity. (d) Higher magnification (×100). Scale bar: 500 *μ*m.

**Figure 4:**
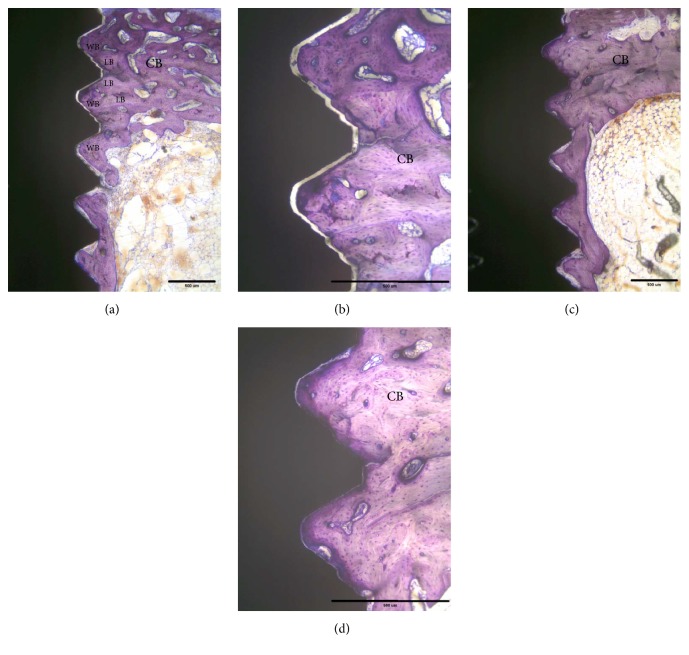
Histological findings of the tested implant after 28 days and 56 days of healing. (a) The implant is surrounded by dense cortical bone (CB) and newly formed bone in the bone marrow cavity. Moreover, woven bone (WB) combined with lamellar bone (LB) was observed in direct contact with the implant surface without the presence of fibrous tissue. Original magnification ×2.5. (b) Original magnification ×10. (c) The implant is surrounded by dense cortical bone (CB) and dense lamellar bone in the bone marrow cavity. (d) Higher magnification (×100). Scale bar: 500 *μ*m.

At sacrifice, none of the implants showed clinical signs of mobility and there was no evidence of bone tissue destruction. All of the retrieved implants showed good osseointegration and were surrounded by bone tissue. Overall, newly formed bone continuously grew in cortical and bone marrow regions after implantation. Initially, the implant was only in partial contact with the original cortical bone (Figures 3(a) and 3(b)). After 14 days of healing, new formation of woven bone was observed both in cortical and in bone marrow regions, as shown in Figures 3(c) and 3(d). The original cortical bone could be clearly identified by its compact and lamellar appearance. The bone-to-implant integration appeared to be primarily a result of ingrowth of bone from the surrounding bone regions. At day 28, woven bone combined with lamellar bone was observed in direct contact with the implant surface without the presence of fibrous tissue (Figure 4(a)). In the cortical regions, the implant was almost surrounded by dense lamellar bone (Figure 4(b)). Finally, at day 56, marked signs of remodeling within the threads were observed both in cortical and in bone marrow regions (Figures 4(c) and 4(d)). No loosened bone debris was observed in the bone marrow regions and there was no cortical bone resorption in the cortical bone regions.

**Figure 5:**
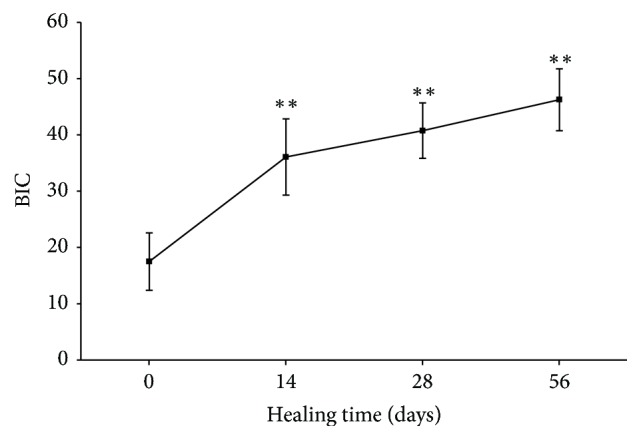
The healing curve plotted with BIC values (^*∗∗*^*p* < 0.01).

As shown in Figure 5, the mean BIC value increased steadily during the healing period. The mean BIC value increased significantly from 17 ± 5.0% at day 0 to 36 ± 6.7% at day 14. The BIC values remained stable at day 28 (40 ± 4.9) and day 56 (46.2 ± 5.5). There were statistically significant differences in the BIC at days 14, 28, and 56 (*p* < 0.001) compared with the initial BIC. No significant difference was observed between groups at day 28 and day 56.

### 3.4. Correlations between Implant Stability Parameters and Osseointegration Performance

**Figure 6:**
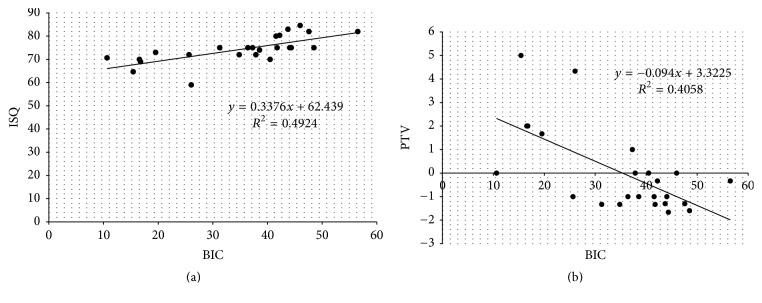
(a) Correlation between implant stability quotient (ISQ) and bone-to-implant contact (BIC) (*R*^2^ = 0.4924, *r* = 0.701, *p* < 0.001). (b) Correlation between PTV values and bone-to-implant contact (BIC) (*R*^2^ = 0.4058, *r* = −0.637, and *p* < 0.05).

**Figure 7:**
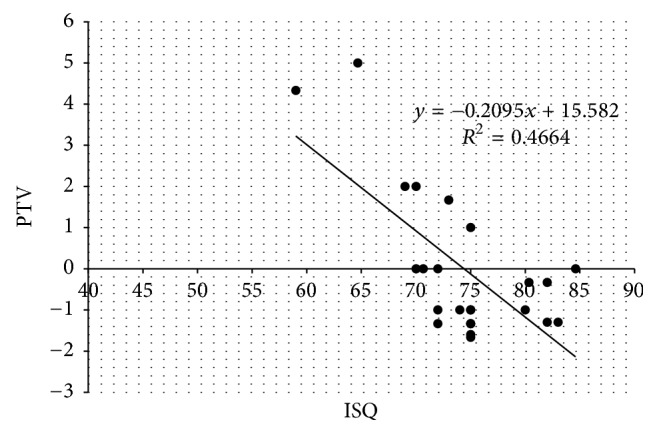
Correlation between implant stability quotient (ISQ) and PTV values (*R*^2^ = 0.4664, *r* = −0.68, and *p* < 0.05).

As shown in Figures 6(a) and 6(b), statistically significant correlations were found between ISQ and BIC (*n* = 24, *R*^2^ = 0.4924, *r* = 0.701, and *p* < 0.001) and PTV and BIC (*n* = 24, *R*^2^ = 0.4058, *r* = −0.637, and *p* < 0.05). Further, it was noted that PTV values showed a more irregular distribution than ISQ values. In addition, there was also a moderate correlation between ISQ and PTV (*n* = 24, *R*^2^ = 0.4664, *r* = −0.68, and *p* < 0.05) as shown in Figure 7.

## 4. Discussion

It is important to quantitatively evaluate implant stability and osseointegration; however, many of the diagnostic methods are invasive such as the removal torque test and the push-out/pull-out test. Although the ISQ and PTV are widely used to noninvasively monitor implant stability, their associations with histomorphometric data during the healing process are still controversial and have not been definitively established [8, 21–23]. Therefore, the present study investigated whether implant stability parameters (ISQ and PTV values) correlated with peri-implant-bone healing (osseointegration, BIC values) at various healing time points. In addition, the correlations between ISQ and PTV values were also evaluated.

RFA can be accomplished without disturbing the process of osseointegration of dental implants during the experimental period. As shown in Figure 2(a), the ISQ values increased after 14 days of healing and remained stable from day 14 to day 28. In addition, the ISQ values were significantly higher at day 56 than at the other time points. The ISQ values representative of successful osseointegration are reported to range from 57 to 82 [30]. In the present study, the measured ISQ values ranged from 67 at day 0 to 81 at day 56, indicating that all of the implants had adequate primary and secondary implant stability. In addition, the healing curve of ISQ values throughout the study period corresponded well with that reported in a previous animal study [25].

Histologic and histomorphometric assessment is the most accurate method to evaluate morphological changes at the bone-implant interface. In this present study, a good bone tissue response to the implant surface was observed during the healing process (Figures 3 and 4). These histological findings were consistent with the results of histomorphometric data (Figure 5). Moreover, the BIC measurements showed a gradual increasing trend throughout the study period and reached 46.2% after 56 days. These BIC values after 56 days of healing were comparable to previous findings in the same animal model [31, 32]. As shown in Figures 3 and 4, woven bone combined with lamellar bone was observed in direct contact with the implant surface without the presence of fibrous tissue, although small microgaps were noted between the bone and the implant. Microgaps occur because of the large difference in the elastic modules between bone and the implant. The sawing, grinding, and polishing procedures during preparation of the ground sections may easily cause detachment of bone from the implant surface. Microgaps in the histological figures were seen in previous studies [23, 33, 34].

We also found that the healing curve of ISQ values was markedly similar to that plotted by BIC values (Figure 5). In addition, a statistically significant positive correlation between ISQ values and BIC (*n* = 24, *r* = 0.701, *p* < 0.001) was demonstrated (Figure 6(a)). These results are consistent with a number of studies that demonstrated a statistically significant correlation between ISQ values and BIC values [2–29, 35]. Nkenke et al. found a positive correlation between RFA and BIC in human cadaver bone using stepped cylinder implants [27]. In addition, three animal studies and one clinical study also reported a strong positive correlation between the two values [22, 28, 29, 35]. However, weak correlations between ISQ values and histomorphometric data have been demonstrated in other animal and human cadaver studies [8, 21, 23–26]. These differences could be explained by the fact that RFA measurements can be influenced by several factors, such as implant diameter, implant surface treatments, bone density, thickness of cortical bone, and surgical technique [36–39]. Moreover, the study designs of the above-motioned studies differ in their selection of animal models, surgical techniques, and implant types. Similarly, Hernández-Cortés et al. found no associations between initial ISQ values and histomorphometric results in human femoral heads [40]. This is because primary implant stability measured by ISQ values was significantly correlated with the cortical bone thickness, but not with bone histomorphometric parameters [23, 37, 41]. However, peri-implant healing in both the cortical and bone marrow regions is important for success of dental implants and also contributes to increasing ISQ values [42]. Therefore, in this present study, both primary and secondary implant stability were measured and associated with histomorphometric data from various healing time points to eliminate the influence of the cortical bone effect.

The Periotest device was developed to evaluate tooth mobility. The time required for an electronically driven rod to make contact with the object surface reflects the degree of implant stability caused by osseointegration [43]. Chavez et al. found that the PTVs of 56 clinically successful dental implants ranged from 2 to −6 [44]. In the present study, the PTVs ranged from 2.5 to −0.7, indicating that all measured implants were stabilized by the surrounding bone. Moreover, a statistically significant positive correlation between PTVs and BIC was demonstrated (Figure 6(b)). This finding is in agreement with previously reported findings [18, 27, 28]. In addition, the Periotest device is a clinically versatile and precise modality for evaluating mini-implant stability [45]. In a recent animal study, Inaba reported a strong correlation between PTVs and BIC ratio [46]. In contrast, Jun et al. reported that there was no significant correlation between PTVs and BIC values in a human fresh cadaver study [21]. They also suggested that the tested implant stability parameters do not seem to be a reliable means of predicting bone-to-implant contact initially after implant placement. Their finding can be explained by the fact that measurement of PTVs is easily affected by the direction, distance, and angle of the tapping head. A variation of 2.5 to 4.0 of PTVs according to the measuring angle relative the healing abutment was reported in a human bone specimen [47]. Moreover, the force of repeated tapping measurements at the time of implant placement may damage the bone-implant interface, especially in implants placed in low-quality bone [48]. We also found that the healing curve of PTVs was more similar to the healing curve plotted by BIC values than to the ISQ healing curve (Figure 5). However, there were larger variations in the Periotest analysis than in the RF analysis at different time points. From a clinical viewpoint, using Periotest is more convenient because the suprastructures of the dental implants need not be removed when performing these measurements.

ISQ values and PTVs can indirectly indicate the degree of osseointegration during the healing period. However, the designs and operating procedures of the two devices that measure those values are quite different. In the present study, we found a significant correlation between ISQ and PTV measurements (Figure 7, *p* < 0.05). These results correspond with those reported in previous studies [28, 49], in which ISQ values correlated with PTVs and both methods were suggested to be comparably reliable. In a finite element analysis study, Winter et al. evaluated the influence of parameters including implant length, bone quality, bone loss, and quality of transducer fixation on RF analysis and Periotest measurements. Good correlation between the two devices was observed only when measurement values of implants with no bone loss were considered [50]. In a clinical study, Oh and Kim evaluated primary implant stability using the Periotest device and the Osstell RFA system and found that the measurements obtained from both devices are associated with bone quality type [51]. Zix et al. demonstrated moderate-to-good correlation between the Periotest device and the Osstell RFA system in a controlled clinical trial. They reported that Periotest was more susceptible to clinical conditions and RF analysis appeared to be a more precise technique [52]. These results demonstrated evidence that monitoring changes in the ISQ and PTV values during the implant healing process can provide valuable information on osseointegration. In the clinic, both ISQ and PTV values can be determined to assess implant stability and the healing status at the bone/implant interface to avoid early implant failures. Considering the limitations of this present study, large sample sizes and controlled clinical studies are needed to validate these viewpoints.

## 5. Conclusion

Based on the results of the present study, it can be concluded that implant stability parameters correlate positively with histomorphometric data during the healing process, indicating that both ISQ and PTV values are useful for measuring implant stability and are reliable for indirectly predicting the degree of osseointegration.
